# Slowly exchanging bound states of SARS-CoV-2 3CL^pro^-inhibitor complexes revealed by ^19^F NMR

**DOI:** 10.1063/4.0001210

**Published:** 2026-08-20

**Authors:** Anna De Falco, Ben Shurina, Rebecca Greene-Cramer, Theresa A. Ramelot, Gaetano T. Montelione

**Affiliations:** Department of Chemistry and Chemical Biology, Center for Biotechnology and Interdisciplinary Sciences, Rensselaer Polytechnic Institute, Troy, New York 12180, USA

## Abstract

The SARS-CoV-2 virus causes COVID-19, and several of its gene products have been successfully targeted for antiviral drug development, including the 3C-like protease (3CL^pro^). The substrate-binding site of 3CL^pro^ exhibits significant conformational plasticity. While available X-ray crystal structures reveal substantial loop variability and molecular dynamics simulations indicate that conformational heterogeneity persists in ligand-bound complexes, the slow-timescale thermodynamic and kinetic landscape of these complexes in solution remains incompletely defined. Using ^19^F NMR spectroscopy, we characterize a slow-exchange conformational equilibrium in both the covalent nirmatrelvir (NMV) and noncovalent ensitrelvir (ENS) complexes of 3CL^pro^. For the wild-type 3CL^pro^–NMV complex at 298 K, joint dual-field line shape and exchange spectroscopy analysis resolves a millisecond-timescale exchange between two distinct states (population ratio ∼ 65:35; *k_ex_* ≈ 52 s^−1^; ΔG^‡^ ≈ 15 kcal mol^−1^). Co-existing bound states also persist in both the noncovalent NMV–[C145A] mutant (minor state population, *p_B_* ≈ 18%) and wild-type ENS (*p_B_* ≈ 38%) complexes, demonstrating that this slow-exchange heterogeneity is an intrinsic property of the ligated protease rather than a consequence of covalent attachment. These dynamics are not readily explained by available crystal structures or conventional microsecond molecular dynamics simulations, suggesting that in solution the inhibited enzyme samples alternative, energetically accessible conformations that are not fully represented in the crystal lattice. This study highlights the utility of solution-state ^19^F NMR for quantifying low-energy conformational states relevant to drug–target energetics and inhibitor design.

## INTRODUCTION

Coronavirus infectious disease 2019 (COVID-19) is caused by severe acute respiratory syndrome coronavirus 2 (SARS-CoV-2), a single-stranded RNA virus of the *Coronaviridae* family.^1–3^ Several SARS-CoV-2 gene products have been targeted for antiviral drug development, including its 3C-like protease (3CL^pro^, also known as the “main protease,” M^pro^). This essential cysteine protease cleaves the viral polyproteins pp1a and pp1ab at over eleven conserved sites,^4^ releasing nonstructural proteins needed for replication and transcription.

**FIG. 1.**
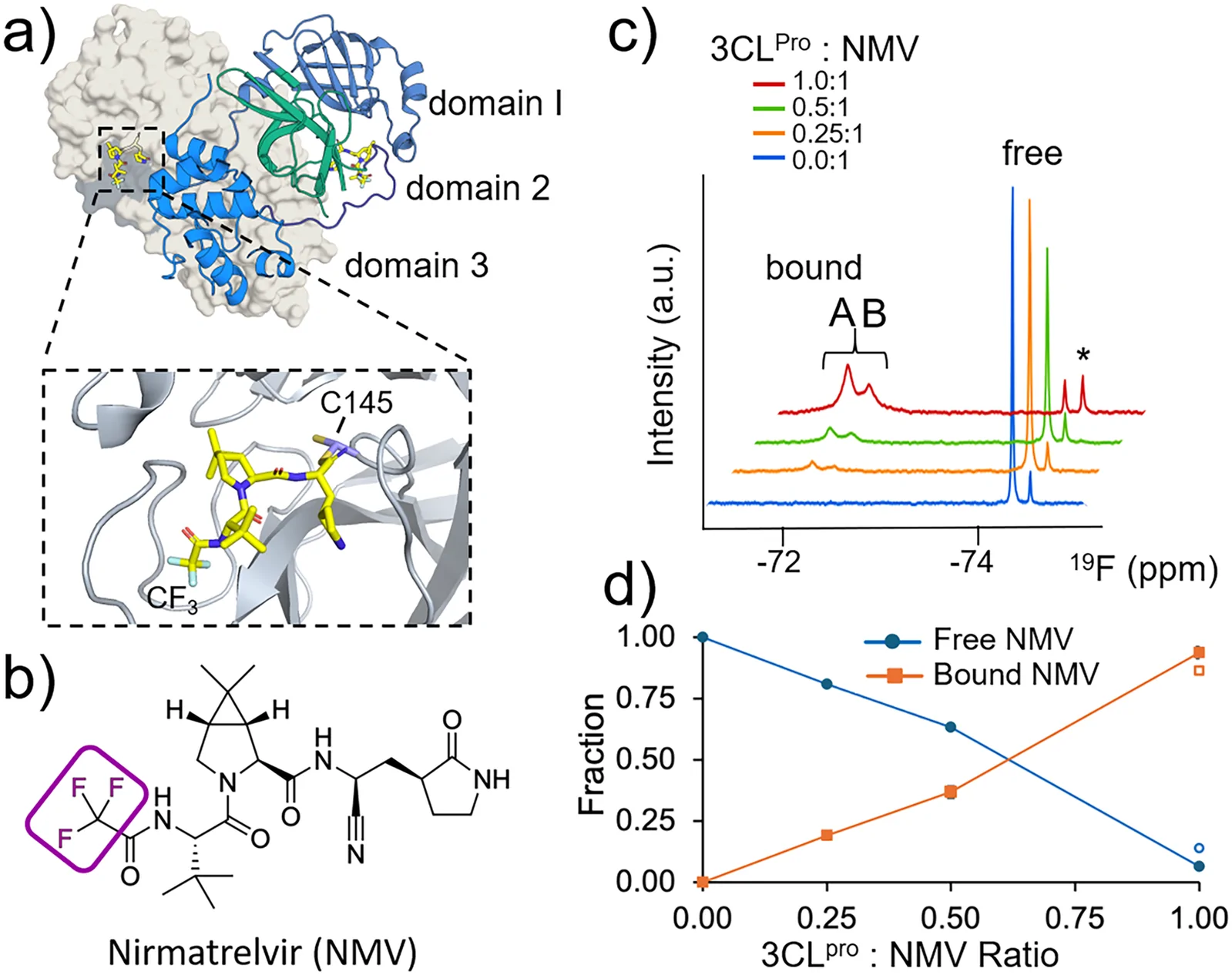
Nirmatrelvir binds SARS-CoV-2 3CL^pro^ with 1:1 stoichiometry and exhibits conformational heterogeneity. (a) X-ray crystal structure of the SARS-CoV-2 3CL^pro^ dimer complexed with NMV (PDB: 7VH8). (b) Chemical structure of NMV containing a trifluoromethyl (–CF_3_) probe. (c) 1D ^19^F NMR titration of 160 μM NMV with increasing concentrations of 3CL^pro^ at 298 K recorded at 600 MHz. Spectra show the depletion of the sharp free ligand resonance and the concomitant emergence of two broad bound-state resonances (A and B) in the slow-exchange regime. An asterisk denotes a non-interacting impurity. (d) NMR binding isotherm. Normalized integrated intensities of the free NMV (blue circles) and the total bound species (A and B, orange squares) plotted against the 3CL^pro^ (single protomer): NMV molar ratio. The linear increase in bound signal and its saturation near the equimolar ratio confirm high-affinity binding with 1:1 stoichiometry. Free and bound fractions for the 1:1 sample at 10 days after preparation are shown as open symbols.

The mature 3CL^pro^ enzyme is a homodimer, for which each protomer consists of three domains [Fig. 1(a)].^5,6^ Domains I and II form a 3C-like fold that includes the enzyme active site. Domain III mediates dimer formation. Catalysis involves a conserved Cys145–His41 dyad. Its substrate-binding and catalytic active sites are characterized by subsites designated S1, S2, S3, S4, and S1–3′, which bind residues at positions P1, P2, P3, P4, and P1–3′, respectively, in polypeptide substrates.^6^ Each subsite is composed of residues from multiple loops nearby the active site. The S1 subsite, defined by Phe140, Asn142, His163, Glu166, and His172 of protomer A and Ser1 of protomer B,^6^ ensures specificity for glutamine at substrate position P1. Notably, the N-terminus of one protomer stabilizes the active conformation in the other protomer through interactions involving Glu166, linking active-site organization to dimer integrity. The S2 subsite, formed by His41, Met49, and Tyr54, accommodates bulky aliphatic side chains at substrate position P2.^6^ The homodimer is relatively stable, with *K*_d_ ∼ 10 μM.^5,7,8^

Several studies have demonstrated conformational plasticity of 3CL^pro^, particularly in the enzyme active site. Room-temperature X-ray crystallography of apo-3CL^pro^ shows that the loops flanking the active site are highly flexible, conferring inherent plasticity in the substrate-binding cavity,^9^ and NMR studies report resonance broadening and multiple peaks for some residues in and around the active-site,^10–12^ suggesting that this plasticity causes intermediate- and slow-timescale conformational exchange. Molecular dynamics simulations of both apo and ligand-bound 3CL^pro^ also detect fast timescale subsite rearrangements of loops that compose the active site.^13,14^ Together, these results indicate that 3CL^pro^ recognizes ligands within a dynamic active site and demonstrate the importance of its flexibility in inhibitor interactions.

Nirmatrelvir (NMV) is an orally bioavailable peptidomimetic inhibitor of 3CL^pro^. It forms a reversible covalent thioimidate adduct between its nitrile warhead and the catalytic Cys145 residue. This interaction leads to high potency and prolonged target residence time with an IC_50_ of ∼20 nM, and a K_i_ of ∼3 nM.^15^ The combination of NMV with ritonavir, a CYP3A inhibitor, is marketed for clinical use as Paxlovid^TM^.^16^ Ensitrelvir (S-217622, ENS) is another orally bioavailable, non-covalent, non-peptidic inhibitor of 3CL^pro^. It binds reversibly, with an IC_50_ of ∼50 nM.^17^ Rather than forming a covalent bond with the catalytic Cys145 residue, ENS engages the S1, S2, and S1′ subsites through hydrogen bonds and hydrophobic interactions.^18,19^ High-resolution crystal structures reveal that the 1-methyl-1H-1,2,4-triazole moiety of ENS forms a key hydrogen bond with His163.^18^ Upon complex formation, these stabilize its interactions with the oxyanion hole and promote a rotated conformation of His41, which in turn makes π–π stacking interactions with the 2,4,5-trifluorophenyl group.^17,19^

Both NMV and ENS contain fluorine atoms in a trifluoroacetyl group and a trifluorophenyl group, respectively. These serve as sensitive, non-perturbing ^19^F NMR reporters of the local protein environment and structural dynamics within these covalent and non-covalent complexes. In both complexes, the ^19^F NMR signals reveal that the drugs do not lock the enzyme into a single static pose. Instead, these complexes populate two conformational states in an approximate 65:35 ratio. This observation of distinct bound resonances is consistent with a ligand sampling two slowly interconverting environments within the binding pocket.^20–22^ These structural dynamics contribute entropically to binding free energies and may be relevant for inhibitor optimization. However, the structural basis of these slowly interconverting states of these complexes is not easily interpretable from available X-ray crystal structures. These studies demonstrate the power of ^19^F NMR for probing conformational plasticity of fluorinated enzyme–inhibitor complexes and the features of these dynamics for two clinically approved drugs.

## RESULTS

### Characterization of the covalent 3CL^pro^–NMV complex

X-ray crystal structures reveal a homodimer with one inhibitor molecule covalently bound to Cys145 within the active site of each protomer [Fig. 1(a)]. To confirm this specificity and stoichiometry in our solution state experiments, we employed high-resolution ESI-Orbitrap mass spectrometry. The mass spectrum (MS) of wild-type 3CL^pro^ revealed an observed mass of 33, 795.4 ± 3.7 Da (calculated 33,796.6 Da). Upon incubation with NMV, a distinct mass shift to 34,296.6 ± 3.2 Da was observed, resulting in a total shift of +501.2 ± 4.9 Da (calculated 499.5 Da), consistent with a 1:1 covalent adduct per protomer. The C145A mutant showed no such modification under identical conditions (observed 33,761.4 ± 2.3 Da; calculated 33,764.6 Da), confirming exclusive covalent modification of Cys145.

To probe the solution properties of the complex, we employed ^19^F NMR spectroscopy using the inhibitor's trifluoromethyl group as a probe [Fig. 1(b)]. Unexpectedly, the ^19^F spectrum of the bound ligand reveals two distinct, broad resonances (designated as arising from states A and B) in slow exchange [Fig. 1(c)], indicating conformational heterogeneity within the active site. Titration experiments demonstrate that the total bound signal intensity saturates at a 1:1 ligand-to-protomer (protein monomer) ratio [Fig. 1(d)], consistent with the stoichiometry observed by mass spectrometry and crystallography. The population ratio of states A and B remains invariant throughout the titration, indicating that these resonances arise from an intrinsic conformational equilibrium within each protomer's active site, rather than multiple binding sites with different affinities, allosteric interactions between sites, or binding to distinct oligomeric states.

Because 3CL^pro^ can bind Zn^2+^ at the active site, coordinated by Cys145 and His residues, metal occupancy was assessed using a previously established ^1^H NMR assay.^23^ In the absence of EDTA, two peaks were observed in the upfield methyl region, corresponding to metal-free and metal-bound states [supplementary material Fig. S1(a)]. These are distinct from the Zn-bound resonance reported previously, and collapse to a single metal-free resonance upon addition of 2 mM EDTA [supplementary material Fig. S1(a)]. Comparison of ^19^F spectra of the NMV-bound complex before and after EDTA addition revealed the same two ^19^F NMR resonances of bound states A and B, with a 10–15% reduction in linewidth [supplementary material Fig. S1(b)], indicating that partial metal occupancy contributes to line broadening. All subsequent NMR experiments were therefore performed in the presence of 2 mM EDTA.

### Equilibrium thermodynamics of NMV-bound conformational states

**FIG. 2.**
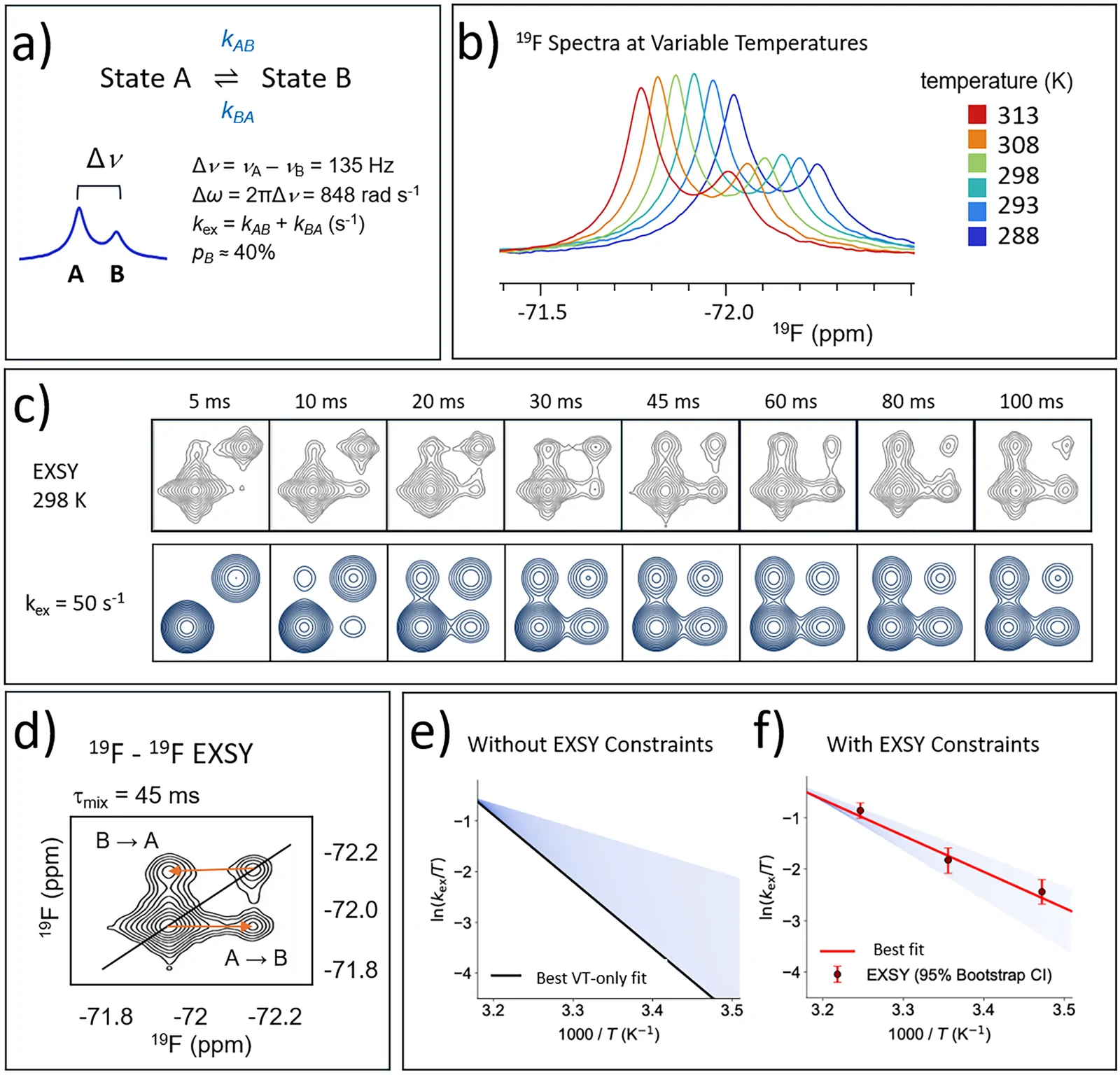
Millisecond conformational exchange of the 3CL^pro^–NMV complex. (a) Two-state exchange model between NMV-bound conformers A and B. (b) Variable-temperature ^19^F spectra at 600 MHz. (c) 2D ^19^F– ^19^F EXSY spectra acquired at 298 K at 600 MHz over a series of mixing times (top) with corresponding simulated spectra using a two-site Bloch–McConnell model (bottom). (d) Representative EXSY spectrum (τ_mix_ = 50 ms) showing exchange cross-peaks between states A and B. (e) and (f) Eyring plots showing ensembles of linear acceptable solutions for the temperature dependence of *k_ex_* derived from dual-field line shape analysis. Thin blue lines represent individual acceptable fits, illustrating the range of Eyring parameters compatible with the data. Panel (e) shows fits obtained using VT line shape data alone, without EXSY constraints. Panel (f) shows fits obtained with incorporation of EXSY-derived constraints.

Two broadened ^19^F resonances [Figs. 1(c) and 2(a)] suggest chemical exchange occurring on the slow-to-intermediate timescale. Variable-temperature (VT) spectra (285–313 K) show minimal changes in line shape, with only slight broadening at the highest temperatures [Fig. 2(b)], consistent with exchange remaining near the slow-exchange regime throughout most of the temperature range. However, prominent ^19^F–^19^F exchange spectroscopy (EXSY) cross-peaks observed across multiple mixing times (τ_m_ = 5–100 ms) confirm millisecond-timescale chemical exchange [Figs. 2(c) and 2(d)].

Global two-state Bloch–McConnell analysis of the VT lineshapes alone did not converge, reflecting the limited sensitivity of the spectra to exchange in this regime. Incorporation of 800 MHz data improved the stability of linewidth estimates, but exchange and relaxation contributions remained partially degenerate. To minimize exchange-induced bias, equilibrium populations were determined from low-temperature spectra in which exchange contributions to linewidths were estimated to be minimal (<10%; supplementary material Fig. S2, Table S1).

The minor-state population (*p_B_*) increases with temperature [supplementary material Fig. S3(a)], indicating that state A is enthalpically favored, while state B is slightly entropically favored. Van't Hoff analysis yields a well-constrained free energy difference at 298 K (ΔG° = +0.33–0.35 kcal mol^−1^), while ΔH° and ΔS° exhibit substantial enthalpy–entropy compensation and are therefore less precisely determined [supplementary material Figs. S3(b) and S3(c)]. Acceptable solutions span ΔH° = +1.1–1.7 kcal mol^−1^ and ΔS° = +2.5–4.7 cal mol^−1^ K^−1^, while maintaining a narrow distribution of ΔG° values [supplementary material Figs. S3(c) and S3(d)].

### Kinetic modeling of millisecond-timescale exchange

The chemical shift separation between states (Δ*ν* ≈ 135 Hz at 600 MHz) places the system at the boundary between slow and intermediate exchange, precluding standalone 1D VT line shape analysis from uniquely determining exchange rates. Under these conditions, multiple Bloch–McConnell parameter combinations provided similarly good fits to the experimental data. Profile likelihood analysis of VT lineshapes alone reveals a broad, poorly constrained solution space [supplementary material Figs. S9(a), S9(c), and S9(e)] and a broad ensemble of acceptable Eyring trajectories [Fig. 2(e)].

To constrain this broad solution space, estimates of exchange rates from independent multi-temperature ^19^F–^19^F EXSY experiments [Fig. 2(c); supplementary material Figs. S4–S6] and ^19^F longitudinal relaxation rates T_1_ were used to constrain the line shape analysis of exchange kinetics. Quantitative analysis of the EXSY buildup curves define well-bounded exchange rates, yielding *k_ex_* (288 K) = 20–32 s^−1^, *k_ex_* (298 K) = 37–61 s^−1^, and *k_ex_* (308 K) = 112–151 s^−1^ (supplementary material Fig. S7). Incorporation of these EXSY-derived constraints on *k_ex_* into the dual-field Bloch–McConnell analysis yields a self-consistent kinetic model that reproduces both the VT lineshapes and EXSY kinetics (supplementary material Fig. S8). The resulting solution ensemble yields *k_ex_* (298 K) = 52 s^−1^ (34–68 s^−1^) and a narrowly defined activation free energy, ΔG^‡^ (298 K) = 14.9–15.4 kcal mol^−1^, while acceptable solutions define broader ranges of ΔH^‡^ = +12–19 kcal mol^−1^ and ΔS^‡^ = −10 to +12 cal mol^−1^ K^−1^, reflecting substantial enthalpy–entropy compensation [supplementary material Figs. S9(b), S9(d), and S9(f)]. Notably, incorporation of EXSY-derived constraints restricts the solution space, yielding a narrower ensemble of acceptable Eyring trajectories consistent with the EXSY-informed kinetic model [Fig. 2(f)].

### Effect of covalent linkage: Analysis of the C145A mutant

**FIG. 3.**
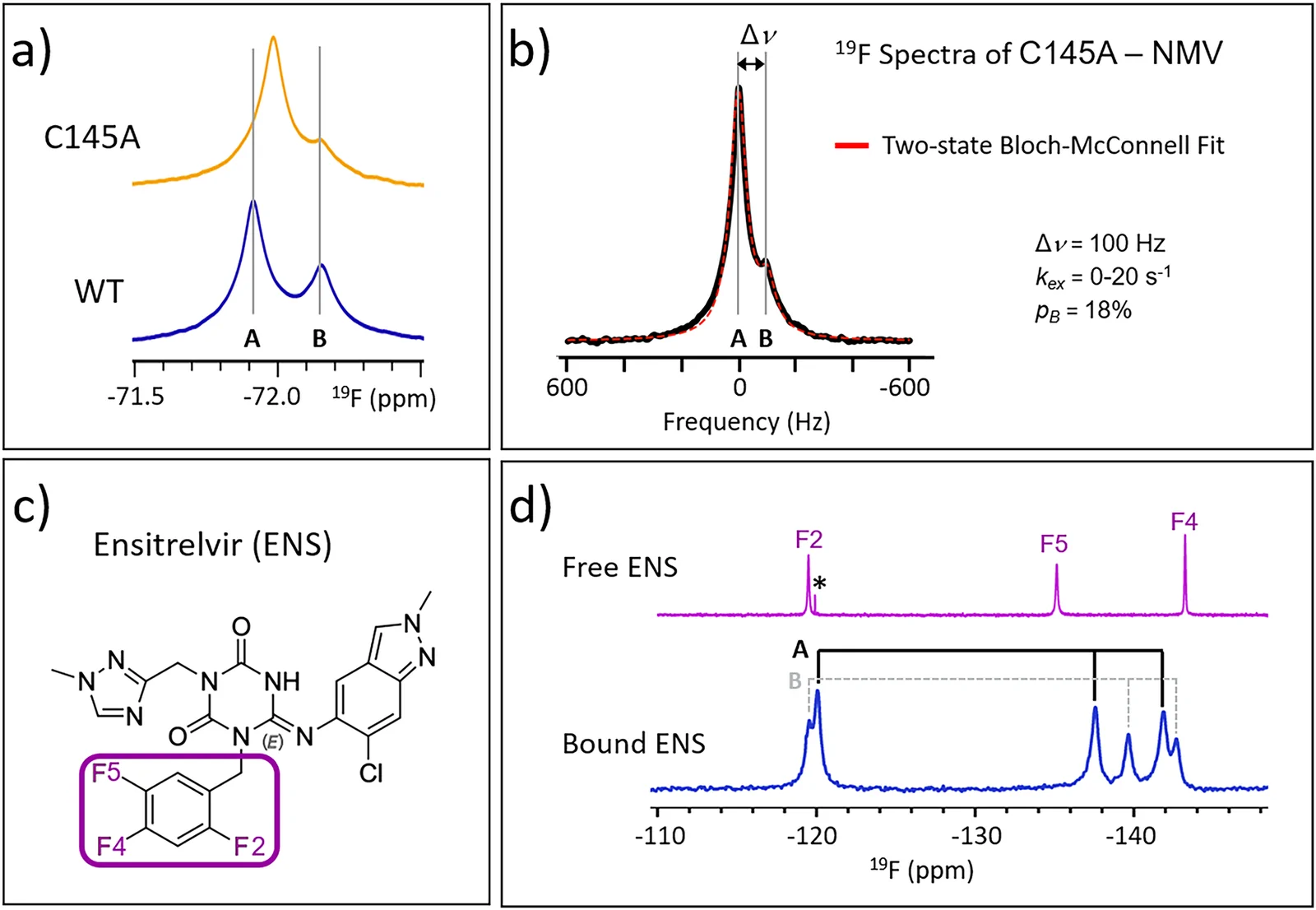
Multiple bound-state environments do not require covalent binding. (a) ^19^F NMR spectrum of NMV bound to [C145A]-3CL^pro^ at 298 K and 600 MHz, showing two bound-state resonances with state A shifted upfield relative to wt 3CL^pro^. (b) Lineshape analysis of the C145A spectrum using a two-state Bloch–McConnell exchange model. (c) Chemical structure of the noncovalent inhibitor ENS with the 2,4,5-trifluorophenyl group highlighted. (d) One-dimensional ^19^F NMR spectra of ENS at 298 K and 600 MHz in the free state and bound to 3CL^pro^, showing two states for each of the three peaks. An asterisk denotes the resonance of trace amounts of F^−^ (−119.5 ppm).

To determine whether the slow-exchanging bound-state heterogeneity requires covalent attachment, we also examined the complex formed between NMV and the C145A active-site mutant. Its ^19^F NMR spectrum at 298 K also exhibits two bound-state resonances [Fig. 3(a)], demonstrating that heterogeneity persists even in the absence of covalent linkage. Relative to wild-type 3CL^pro^, resonance A is shifted upfield, indicating that the mutation alters the local environment without eliminating the two-state behavior. The spectrum is well described by a two-state exchange model analogous to that used for the wild-type complex [Fig. 3(b)]. Using intrinsic linewidths determined from wild-type data, line shape analysis of the C145A spectrum at 298 K was consistent with a slow exchange rate (*k_ex_* ∼ 0 to 20 s^−1^) and *p_B_* ∼ 18% at 298 K. NMV binds to C145A with a reported *K*_d_ of ∼3 μM;^24^ at the 400 μM protein concentration used here >99% of the enzyme is therefore expected to be ligand-bound. These results indicate that covalent attachment is not required to generate multiple bound-state environments and indicate that the two states observed for wild-type 3CL^pro^ do not arise from a mixture of covalently and non-covalently bound NMV or from a mixture of bound and free inhibitor.

### Conformational equilibrium of the noncovalent 3CL^pro^-ENS complex

To determine whether bound-state heterogeneity extends to other inhibitor classes with distinct chemical scaffolds, we evaluated the conformational equilibrium of the noncovalent ENS complex [Fig. 3(c)]. At 298 K, the ^19^F NMR spectrum reveals three resonances arising from the free trifluorophenyl group that, upon binding to 3CL^pro^, resolve into split resonances designated as states A and B [Fig. 3(d)], with a minor state population (*p_B_* ∼ 38%; ΔG° ∼ +0.3 kcal mol^−1^) highly comparable to the NMV complex.

VT spectra (285–313 K) demonstrate that this equilibrium is largely temperature-insensitive, with both states remaining in the slow exchange regime (supplementary material Fig. S3). Unlike the NMV-bound complex, no exchange-induced progressive line broadening was observed upon heating. Instead, linewidths decreased modestly with increasing temperature, due to intrinsic relaxation effects. Notably, even the resonance with the smallest chemical shift separation (Δ*ν* ≈ 300 Hz) remained resolved at the highest temperatures, qualitatively constraining *k_ex_* to values well below this frequency difference (<100 s^−1^).

State populations at each temperature were determined from two-Lorentzian line shape fits to the central resonances and analyzed via a van't Hoff relationship to obtain equilibrium thermodynamic parameters (supplementary material Fig. S10). As observed for NMV, the ENS-3CL^pro^ parameters exhibit enthalpy–entropy compensation. However, the ENS populations remained flat with increasing temperatures, yielding an enthalpy change near zero (ΔH° = 0 ± 1.8 kcal mol^−1^) and a poorly constrained entropy change (ΔS° = −0.8 ± 4.7 cal mol^−1^ K^−1^). A high-resolution spectrum at 298 K is accurately reproduced by simulation using these parameters (supplementary material Fig. S11). The kinetic and equilibrium thermodynamic parameters describing both the NMV- and ENS-bound complexes are summarized in Table I.

**TABLE I.** Thermodynamic and kinetic parameters for the conformational exchange (State A ⇌ State B) in 3CL^pro^–inhibitor complexes.

Parameter	3CL^pro^–NMV^a^	3CL^pro^-ENS	Interpretation
Intrinsic linewidths (Hz) (298 K, 600 MHz)	60–70	170–220	Broader ENS linewidths reflect reduced rotational averaging relative to the rapidly rotating NMV-CF_3_ group.
Intrinsic linewidths (Hz) (298 K, 800 MHz)	120–130		Consistent with expected field-dependent scaling of 600 MHz linewidths.
**Thermodynamics**			
Minor population (*p_B_*, 298 K)	36% [36%–37%]	38% ± 2%	Conformational populations are similar between the two complexes and are well constrained for NMV by low-temperature line shape analysis and van't Hoff fitting.
ΔG^o^ (298 K) (kcal mol^−1^)	0.33 [0.33–0.35]	0.3 ± 0.1	Free energy differences between states are small and well constrained.
ΔH^o^ (kcal mol^−1^)	1.5 [1.1–1.7]	0.0 ± 1.4	Near-zero ΔH^o^ for ENS reflects weak temperature dependence of *p_B_*; positive ΔH^o^ for NMV indicates state A is enthalpically favored.
ΔS^o^ (cal mol K^−1^)	4.1 [2.5–4.7]	−0.8 ± 4.7	ΔH° and ΔS° are correlated due to enthalpy–entropy compensation and are less precisely determined than ΔG°.
**Kinetics**			
*k_ex_* (298 K) (s^−1^)	52 [34–69]	<100	For NMV, *k_ex_* is moderately constrained by combined VT line shape and EXSY-restrained analysis.
ΔG^‡^ (298 K) (kcal mol^−1^)	15.1 [14.9–15.4]	>16	For NMV, ΔG^‡^ is well-constrained from EXSY-restrained dual-field analysis. ENS lower bound estimated from fully resolved slow-exchange peaks up to 313 K.
ΔH^‡^ (kcal mol^−1^)	14 [12–19]		Broad range reflects enthalpy–entropy compensation and parameter covariance.
ΔS^‡^ (cal mol K^−1^)	−4 [−10 to 12]		Strongly correlated with ΔH^‡^ and varies across acceptable solutions due to compensation effects.
NMR exchange regime	Slow/intermediate	Slow exchange	ENS data had no observable exchange-induced line broadening up to 313 K.

^a^ For the NMV complex, values represent the median of the acceptable solution ensemble with brackets indicating the 95% confidence intervals.

## DISCUSSION

### Conformational dynamics in 3CL^pro^–inhibitor complexes

Although high-resolution crystal structures capture precise 3CL^pro^–inhibitor contact geometries, our solution-state NMR analysis reveals ligated active site dynamics on the millisecond timescale. Crucially, all thermodynamic and kinetic parameters determined here describe the unimolecular exchange between two distinct co-existing bound states (State A ⇌ State B) after the inhibitor has fully docked. Both NMV and ENS complexes occupy a shallow two-state equilibrium that slightly favors state A ( $\Delta {\text{G}}_{\text{AB}}^{o}\approx +0.3\text{ kcal }{\text{mol}}^{-1}$; *p_B_* ≈ 40%) yet are separated by a substantial activation barrier (Δ*G*^‡^ ≈ 15 kcal mol^−1^ for NMV, and higher for ENS). The persistence of two states in the noncovalent NMV-C145A complex (*p_B_* ∼ 18%) and the wild-type ENS complex (*p_B_* ∼ 35%) indicates that the observed conformational equilibrium is observed for both covalent and non-covalent 3CL^pro^-inhibitor complexes, and may reflect an underlying property of the inhibited enzyme [Figs. 3(a) and 3(b)].

### Thermodynamic and kinetic features of the dual-state equilibrium

For both wild-type complexes, low-temperature population analysis yields small positive ΔH^o^ values and small ΔS^o^ values with limited precision due to enthalpy–entropy compensation, consistent with a balanced energetic landscape where subtle shifts in packing or hydrogen bonding networks are roughly compensated for by variations in local mobility and solvent organization. While the exact kinetic barriers differ among systems, all three exhibit slow conformational exchange on the NMR timescale.

For the 3CL^pro^–NMV complex, joint global line shape and EXSY modeling defines a millisecond-timescale barrier (ΔG^‡^ ≈ 15 kcal mol^−1^ and *k_ex_* ≈ 50 s^−1^ at 298 K). Corresponding activation parameters indicate an activation enthalpy of approximately 12–19 kcal mol^−1^ coupled with a poorly constrained activation entropy, reflecting substantial enthalpy–entropy compensation within the acceptable solution ensemble. Although detailed activation parameters could not be determined for the ENS complex (*k_ex_* < 100 s^−1^) or the NMV-bound C145A mutant (*k_ex_* ≈ 0–20 s^−1^ at 298 K), the persistence of slow exchange kinetics in all three systems suggests a related underlying thermodynamic landscape. Interestingly, transient kinetic studies of 3CL^pro^ inhibition by both NMV and ENS demonstrate two-phase binding mechanisms involving an initial weaker binding followed by a slower tighter-binding step,^25,26^ which may be related to this energy landscape.

### Equilibrium thermodynamics in 3CL^pro^–inhibitor complexes

State A is thermodynamically favored by a small free energy difference ( ${∆\text{G}}_{\text{AB}}^{o}\;\approx \;0.3\text{ kcal }{\text{mol}}^{-1}$) and differs only modestly in enthalpy from state B ( ${∆\text{H}}_{\text{AB}}^{o}\;\approx \;1.5\text{ kcal }{\text{mol}}^{-1}\text{for NMV}$). The small accompanying entropy difference ( ${∆\text{S}}_{\text{AB}}^{o}\;\approx \;4\text{ cal }{\text{mol}}^{-1}\;K^{-1}\text{for NMV},\text{ favoring state }B$) further suggests that state B is not a highly disordered ensemble but rather an alternative bound conformation of comparable stability. The two states may differ through subtle loop or side chain rearrangements that slightly alter local hydrogen-bonding or packing interactions, incurring a negligible energetic penalty, despite the substantial activation barrier separating the two states. For the ENS complex, the near temperature independence of the state populations indicates a remarkably flat enthalpic landscape ( ${∆\text{H}}_{\text{AB}}^{o}\;\approx \;0$), suggesting that the structural variations between the co-existing states are largely enthalpically neutral, leaving state A only marginally favored by a small enthalpic or entropic advantage ( ${∆\text{G}}_{\text{AB}}^{o}\;\approx \;0.3\text{ kcal }{\text{mol}}^{-1}$).

### Potential origins of the slow conformational equilibrium

**FIG. 4.**
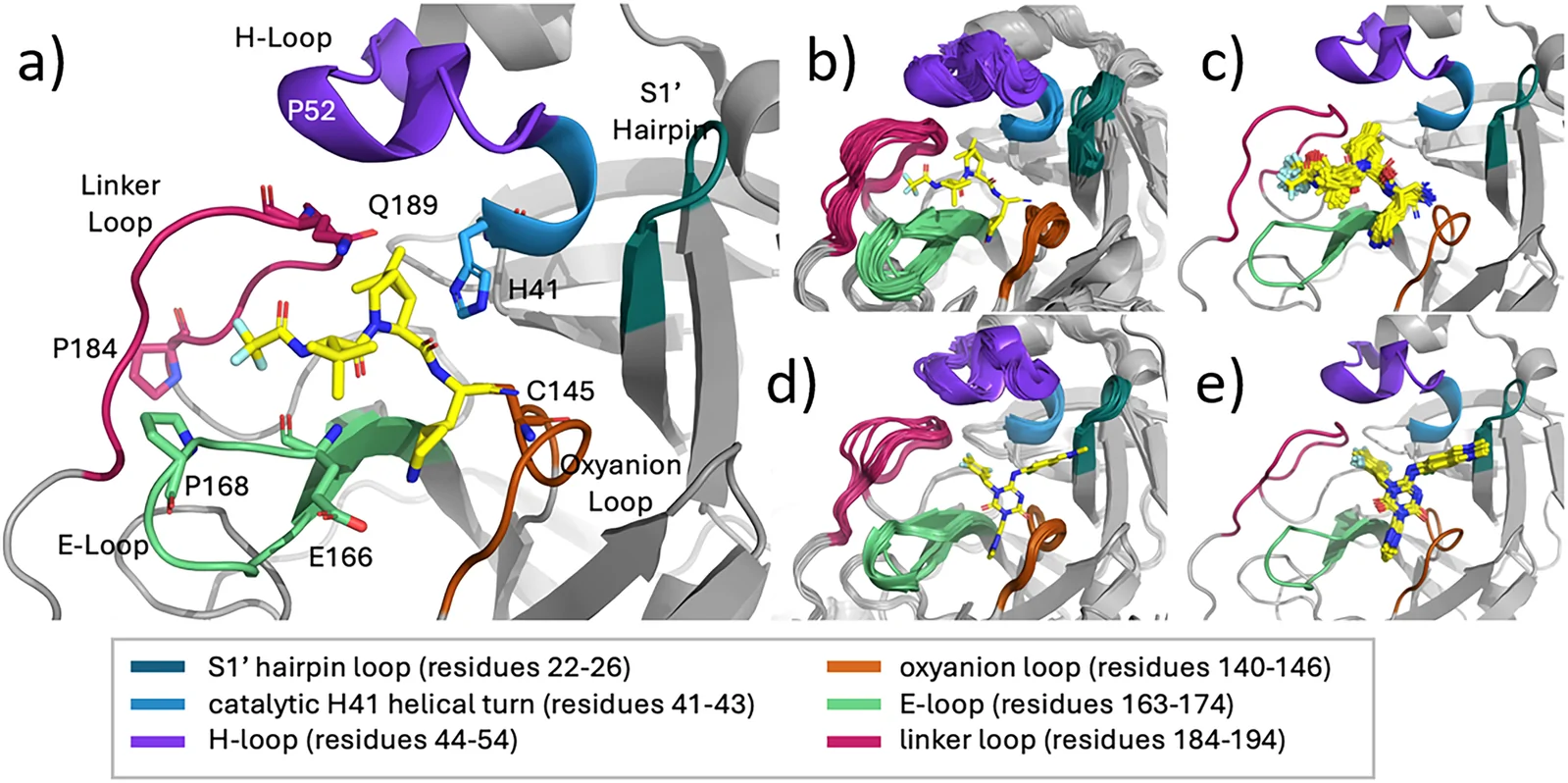
Conformational variations observed in the available X-ray crystal structures of 3CL^pro^–NMV and 3CL^pro^-ENS complexes. (a) Active site of 3CL^pro^ (PDB ID 7VLP_A). Loop regions shown indicate the S1' hairpin loop (residues 22–26, teal), catalytic H41 helical turn (residues 41–43, cyan), H-loop (residues 44–54, purple), oxyanion loop (residues 140–146, orange), E-loop (residues 163–174, green), and linker loop (residues 184–194, magenta), along with the locations of three proline resides (Pro52, Pro168, and Pro184). (b) Variability of relative loop positions in 60 NMV-bound complexes relative to the NMV atoms of 7VLP_A. (c) Variability of NMV pose positions relative to the protein chain from 7VLP_A. (d) Variability of relative loop positions in 23 ENS-bound complexes relative to the ENS atoms of PDB deposition 8HEF. (e) Variability of ENS poses positions relative to the protein chain from 8HEF.

To investigate the structural origins of this slow-timescale motion, we made comparisons across available high-resolution crystal structures of 3CL^pro^–NMV and 3CL^pro^–ENS complexes (listed in supplementary material Table S2). Both complexes are stabilized by dense protein–ligand interaction networks. For NMV, the trifluoroacetamide (–NHCOCF_3_) moiety forms hydrogen bonds with the sidechains of Gln189 and Gln192,^27,28^ and stabilizing multipolar (F…C=O) interactions between the –CF_3_ group and the backbone carbonyl of Glu166.^27,29^ Similarly, the noncovalent ENS inhibitor engages the S2 subsite via a hydrophobic trifluorobenzylic group,^19^ contacting the sidechains of the catalytic-site His41 and of Gln189 in the linker loop and forming a key hydrogen bond between its triazole moiety and the Glu166 side chain in the E-loop.^19,30^ Because the N-terminal region of one protomer contributes to the active site of the other protomer, active-site geometry is inherently coupled to inter-protomer contacts. However, despite some small variations in loop positions [Figs. 4(a), 4(b), and 4(d)], ligand binding poses [Figs. 4(c) and 4(e)], and other minor structural heterogeneity evident across the crystallographic ensembles (supplementary material Fig. S12), comparisons across these structures do not provide an obvious structural basis for the slow-exchange conformational heterogeneity detected by ^19^F-NMR. Although larger loop motions are observed in microsecond molecular dynamics simulations,^14^ these occur on a much faster timescale than the tens-of-millisecond processes detected by ^19^F NMR.

Peptide bond isomerization, and particularly X-Pro isomerization, is a common source of conformational heterogeneity resulting in slow chemical exchange.^31–37^ Candidate residues near the active site include Pro52 in the H-loop, Pro168 in the E-loop, and Pro184 in the linker loop located adjacent to the substrate-binding cleft [Fig. 4(a); supplementary material Fig. S13]. Although all available crystal structures show these residues in the *trans* conformation, NMR studies of apo-3CL^pro^ detected a minor *cis* population of Pro184 (∼10%),^10^ suggesting that proline isomerization remains a plausible source of the observed exchange. In X-ray crystal structures of these complexes, Pro184 is consistently modeled in the *trans* conformation with low crystallographic B factors, and available structures provide no obvious evidence for unusual peptide bond geometry distortion at this position. The activation barrier measured for the NMV complex (∼15 kcal mol^−1^) is somewhat lower than those typically reported for proline *cis*–*trans* isomerization in short linear peptides (16–20 kcal mol^−1^; rates 10–10^−4^ s^−1^),^33^ but significantly reduced barriers have also been reported in cyclic peptides and structured protein environments,^33,35,37,38^ where it may be attributed to stabilization of the isomerization transition state by the surrounding scaffold. At the same time, activation barriers in the range of 13–16 kcal mol^−1^ are also well documented for collective backbone and coordinated loop rearrangements in proteins without requiring peptide bond isomerization,^39,40^ and various alternative mechanisms may underlie the observed slow conformational exchange. Thus, while proline isomerization remains a viable possibility, the present data do not uniquely assign a structural basis to the observed multistate properties of these 3CL^pro^-drug complexes.

## CONCLUSIONS

Beyond the static conformations captured in crystallographic models, our results demonstrate that inhibitor-bound 3CL^pro^ complexes sample multiple ligand-bound states in solution. Using ^19^F NMR, we provide a quantitative view of this thermodynamic landscape, revealing that both covalent and noncovalent inhibitors undergo millisecond-timescale exchange between two states that are similar in free energy, but separated by substantial kinetic barriers. Consequently, the hundreds of available 3CL^pro^–inhibitor structures may represent a restricted view of a more complex solution-state energy landscape. While our study does not resolve the exact mechanism of the slow exchange process, it illustrates the utility of solution-state ^19^F NMR for quantifying hidden, co-existing conformational equilibria relevant to inhibitor design and antiviral drug discovery.

## METHODS

### Production of wild-type and mutant 3CL^pro^

For protein production, plasmids pGTM_CoV2_NSP5_004_SUMO or pGTM_CoV2_NSP5_C145A_SUMO were expressed in *E. coli* BL21 (DE3) cells and purified to obtain 3CL^pro^ (or C145A-3CL^pro^) in a pure and native form, following the detailed protocol previously described by our group.^41^ The resulting protein sample was >98% homogeneous based on SDS-PAGE (Sodium Dodecyl Sulfate–Polyacrylamide Gel Electrophoresis). Protein concentrations were determined by UV-vis spectrophotometry at 280 nm (ϵ = 33,020 M^−1 ^cm^−1^).

### Antiviral drugs

NMV and ENS were purchased from MedChemExpress (Sollentuna, Sweden). Chemical homogeneity was confirmed by 1D ^1^H and ^19^F NMR.

### Mass spectrometry

Wild-type and C145A SARS-CoV-2 3CL^pro^ samples were incubated with a 1:1 molar ratio of NMV overnight at 4 °C. Intact mass spectra were acquired on a high-resolution LTQ Orbitrap XL mass spectrometer, and neutral masses were calculated from assigned deconvoluted charge states using Thermo Xcalibur Qual Browser software.

### NMR sample preparation and spectroscopy

All NMR samples were prepared in 20 mM Tris, 100 mM NaCl, 5 mM DTT, 1 mM TCEP, pH 7.4, containing 10% D_2_O and 2% DMSO. To minimize metal-induced line broadening, 2 mM EDTA was included in all samples unless otherwise noted. ^19^F NMR spectra were acquired on three Bruker spectrometers, an AVIII 600 MHz, a NEO 600 MHz, and a NEO 800 MHz, each equipped with TCI 5 mm solution (H-F/D/C/N) CryoProbes. ^19^F chemical shifts were referenced to the unified chemical shift scale relative to the absolute frequency of the ^1^H lock signal, which places the internal fluoride ion (F^−^) near the expected value of −119.5 ppm. The absolute sample temperature was calibrated using a 100% methanol standard. All spectra were processed in TopSpin 4.0.9 using baseline correction and line broadening appropriate to each experiment. Samples used for EDTA control experiments were prepared identically in the absence of EDTA, followed by addition of 2 mM EDTA or 300 μM Zn^2+^ as indicated (supplementary material Fig. S1).

### Titration of nirmatrelvir with 3CL^pro^

A 160 μM solution of NMV was titrated with a concentrated stock of SARS-CoV-2 3CL^pro^ to achieve protein-to-ligand molar ratios of 0.25, 0.5, and 1.0. 1D ^19^F NMR spectra were acquired on a Bruker AVIII 600 MHz spectrometer at 298 K. Spectra were processed with a 10 Hz exponential line broadening function. Signal intensities were quantified by direct integration of the ^19^F resonances. The free ligand population was determined from the integral of the sharp free ligand resonance (δ_*free*_ = −74.39 ppm). Due to baseline overlap, the bound state populations were quantified by integration of both the A and B resonances as a single composite signal. Binding saturation was evaluated by plotting normalized free and bound integrated intensities against the protein-to-ligand molar ratio.

### Variable-temperature 1D ^19^F NMR spectroscopy

VT ^19^F NMR spectra of the 3CL^pro^–NMV complex were acquired between 285 and 313 K at both 600 and 800 MHz on Bruker NEO spectrometers. Samples contained 400 μM 3CL^pro^ and NMV at a 1:1 molar ratio. Spectra were processed using exponential line broadening of 10 Hz. This additional Lorentzian broadening introduced during processing was explicitly incorporated into all subsequent line shape simulations and fitting procedures. The temperature-dependent spectra revealed two resonances corresponding to slowly exchanging conformational states designated A and B.

### Equilibrium thermodynamic analysis

Intrinsic spectral parameters were obtained from independent Lorentzian fits of the VT ^19^F spectra at each field strength. These fits yielded temperature-dependent linewidths, chemical shift separations (Δ*ν*), and equilibrium populations (*p_A_*, *p_B_*). To minimize exchange contributions, thermodynamic analysis was restricted to spectra in the low-temperature slow-exchange regime. Bloch–McConnell simulations were used to estimate the exchange contribution to the linewidth (LW_ex_), and spectra with >10% exchange contributions to the fitted intrinsic linewidth were excluded (supplementary material Table S1).

Equilibrium constants were calculated as *K_eq_* = *p_B_*/*p_A_* and analyzed using the van't Hoff relationship,

$$K_{eq}\left(T\right)\;=\text{ exp }[-(\Delta H{}^{\circ}-T\cdot \Delta S{}^{\circ})/RT].$$

To evaluate parameter uncertainty and account for covariance, van't Hoff linear regressions were systematically performed across multiple subsets of low-exchange data. Solutions were evaluated based on the residual sum of squares (RSS) of ln(*K_eq_*). An ensemble of acceptable fits was defined using an empirical threshold (RSS ≤ 1.15 × RSS_min_). The filtered solution ensemble was used to map parameter compensation and determine median values and 95% confidence intervals for ΔH°, ΔS°, ΔG° (298 K), and *p_B_* (298 K).

### Two-state Bloch–McConnell line shape analysis

Variable-temperature ^19^F NMR lineshapes acquired at 600 and 800 MHz were analyzed using a two-site Bloch–McConnell exchange model. The temperature dependence of the exchange rate constant was constrained using the Eyring relationship,

$$k_{ex}\left(T\right)\;=\;\left(k_{B}\cdot T/h\right)\;\cdot \text{ exp }[-(\Delta H^{‡}-T\cdot \Delta S^{‡})/RT].$$

Equilibrium populations were parameterized using ΔH° and ΔS° obtained from independent van't Hoff analysis of low-exchange spectra. These parameters were used to calculate temperature-dependent populations (*p_B_*) during Bloch–McConnell fitting, ensuring thermodynamic consistency. To reduce degeneracy between exchange broadening and intrinsic relaxation, the temperature dependence of chemical shift separations (Δ*ν*) and intrinsic linewidths was constrained using independent analyses of the low-temperature slow-exchange regime (supplementary material Fig. S2). Global fitting was performed hierarchically, in which independently parameterized thermodynamics and intrinsic spectral parameters from the slow-exchange regime were used to constrain the dual-field Bloch–McConnell analysis. Remaining free parameters included intrinsic linewidth intercepts, Eyring activation parameters, and overall spectral scaling factors.

To further reduce ambiguity arising from linewidth–exchange compensation, exchange rates obtained from independent EXSY measurements at 288, 298, and 308 K were incorporated into the fitting objective function as soft restraints. Deviations between Eyring-predicted and EXSY-derived exchange rates were weighted by the corresponding EXSY uncertainties, yielding a hybrid fit jointly constrained by VT line shape and EXSY data. This yielded a hybrid model in which VT line shape data and EXSY measurements jointly determine the optimal kinetic solution. To assess parameter identifiability, profile likelihood analysis was performed both for VT line shape fitting alone and for the hybrid VT + EXSY fitting procedure. In each case, ΔG^‡^ (298 K) was systematically varied and remaining parameters re-optimized. Acceptable solutions were defined using a residual sum-of-squares (RSS) threshold relative to the global minimum, yielding ensembles of kinetic trajectories consistent with the experimental data [Figs. 2(e) and 2(f); supplementary material Fig. S9]. The EXSY-constrained solution ensemble was used to estimate confidence intervals and assess enthalpy–entropy compensation (Table I; supplementary material Fig. S9).

### 2D ^19^F–^19^F EXSY data collection and kinetic analysis

Two-dimensional ^19^F–^19^F exchange spectroscopy (EXSY) spectra of the 3CL^pro^–NMV complex were acquired at 288, 298, and 308 K on a Bruker NEO 600 MHz system. Spectra were acquired with a relaxation delay (d1) of 4.0 s using a series of mixing times spanning 5–150 ms and processed in TopSpin 4.0.9 with 15 Hz line broadening and baseline correction.

Exchange cross-peaks between states A and B confirmed chemical exchange on the millisecond timescale. Exchange rates (*k_ex_*) were determined by fitting exchange buildup curves constructed from normalized peak-height ratios, AB/(AA + AB) and BA/(BB + BA), where AA and BB denote diagonal peaks and AB and BA denote exchange cross-peaks. This normalization reduces sensitivity to overall intensity scaling and T_1_ relaxation effects. Buildup curves were fit using two-site Bloch–McConnell simulations, in which spectral parameters independently determined from 1D line shape analysis at each temperature (Δ*ν*, linewidths, and equilibrium populations) were fixed. Longitudinal relaxation rates (*R*_1_ = 1/*T*_1_) were measured at each temperature and incorporated into the exchange model. This approach isolates *k_ex_* as the only adjustable parameter in the EXSY curve-fitting pipeline and for simulations. Exchange rate constants were obtained by nonlinear least squares optimization of the experimental buildup curves. Parameter uncertainties were estimated using residual bootstrap resampling (N = 500), yielding median k_ex_ values and 95% confidence intervals. Additional details of data processing, simulation parameters, and fitting procedures are provided in the supplementary material (supplementary material Figs. S4–S7).

### Analysis of the 3CL^pro^-ENS complex

For the 3CL^pro^–ENS complex, spectra (400 μM, 1:1) were acquired from 288 to 313 K at 600 MHz and processed with 50 Hz additional line broadening. Across this temperature range, linewidths decreased with increasing temperature and no exchange-induced broadening or coalescence was observed, indicating that exchange contributions were not experimentally separable from intrinsic relaxation. Consequently, the data did not provide sufficient sensitivity to reliably extract *k_ex_* via a global Bloch–McConnell or Eyring analysis. Accordingly, spectra were globally analyzed with a coupled two-Lorentzian model. State populations were obtained from the central resonances and used to calculate *K_eq_* as a function of temperature. Thermodynamic parameters were obtained from a van't Hoff analysis. Uncertainties in ΔH*°,* ΔS*°, K_eq_,* and *p_B_* were estimated from the experimental scatter of independent temperature-specific fits, with 95% confidence intervals derived from regression analysis of these data (supplementary material Fig. S10). The smallest observed chemical shift separation (∼300 Hz) for the left resonance provides an experimental upper bound on *k_ex_*. The model was independently validated at 298 K against a high-S/N dataset processed with minimal line broadening (supplementary material Fig. S11).

### Analysis of the [C145A]-3CL^pro^–NMV complex

For the [C145A]-3CL^pro^–NMV complex (400 μM, 1:1), spectra were acquired at 298 K at 600 MHz and processed with 5 Hz line broadening. The resulting 1D ^19^F spectrum was fit to a two-site Bloch–McConnell model with intrinsic linewidths fixed to those determined for the wild-type complex (adjusted for processing broadening), while *k_ex_, p_B_*, and Δ*ν* were optimized by nonlinear least squares analysis.

### Molecular graphics

Protein structures were visualized and rendered using Open PyMOL 3.1.0 (Schrödinger, LLC).^42^ Structural superpositions were performed using the built-in alignment algorithm in PyMOL by aligning the Cα-atoms of residues in domains I and II (residue range 1–199). The full list of PDB entries used in the alignments can be found in supplementary material Table S2.

### Python scripts

All simulations and fitting procedures were implemented using custom Python scripts based on the Bloch–McConnell formalism. The scripts used for line shape fitting, EXSY simulation, and parameter analysis are publicly available as described in the Data Availability section.

## SUPPLEMENTARY MATERIAL

See the supplementary material includes thermodynamic and kinetic characterization via variable-temperature line shape analysis, 2D EXSY validation of the two-state exchange model, and structural comparisons of NMV and ENS complexes, including a comprehensive list of analyzed PDB entries.

## Data Availability

The data that support the findings of this study are openly available in Zenodo at 10.5281/zenodo.20572660, Ref. 43 and GitHub at https://github.com/MontelioneLab/3CLpro-inhibitor-dynamics-19F_analysis, Ref. 44.
